# Identification of critical pathways and potential therapeutic targets in poorly differentiated duodenal papilla adenocarcinoma

**DOI:** 10.1186/s12935-020-01709-7

**Published:** 2021-01-06

**Authors:** Yuanxiang Lu, Wensen Li, Ge Liu, Yongbo Yang, Erwei Xiao, Senmao Mu, Yuqi Guo, Deyu Li, Guoyi Yan

**Affiliations:** 1 Department of Hepatobiliary Surgery, Henan Provincial People’s Hospital, Zhengzhou University People’s Hospital, Zhengzhou, China; 2 School of Clinical Medicine, Zhengzhou University, Zhengzhou, China; 3 School of Clinical Medicine, Henan University, Kaifeng, China; 4 Department of Pharmacy, Zhongmou People’s Hospital, Zhengzhou, China

**Keywords:** Duodenal papilla carcinoma, Biomarker, Differentially expressed genes, RNA-seq

## Abstract

**Background:**

Duodenal papilla carcinoma (DPC) is a rare malignancy of the gastrointestinal tract with high recurrence rate, and the pathogenesis of this highly malignant neoplasm is yet to be fully elucidated. This study aims to identify key genes to further understand the biology and pathogenesis underlying the molecular alterations driving DPC, which could be potential diagnostic or therapeutic targets.

**Methods:**

Tumor samples of three DPC patients were collected and integrating RNA-seq analysis of tumor tissues and matched normal tissues were performed to discover differentially expressed genes (DEGs). Gene Ontology (GO) and Kyoto Encyclopedia of Genes and Genomes (KEGG) enrichment analysis were carried out to understand the potential bio-functions of the DPC differentially expressed genes (DEGs). Protein–protein interaction (PPI) network was constructed for functional modules analysis and identification of hub genes. qRT-PCR of clinical samples was conducted to validate the expression level of the hub genes.

**Results:**

A total of 110 DEGs were identified from our RNA-seq data, GO and KEGG analyses showed that the DEGs were mainly enriched in multiple cancer-related functions and pathways, such as cell proliferation, IL-17signaling pathway, Jak-STAT signaling pathway, PPAR signaling pathway. The PPI network screened out five hub genes including IL-6, LCN2, FABP4, LEP and MMP1, which were identified as core genes in the network and the expression value were validated by qRT-PCR. The hub genes identified in this work were suggested to be potential therapeutic targets of DPC.

**Discussion:**

The current study may provide new insight into the exploration of DPC pathogenesis and the screened hub genes may serve as potential diagnostic indicator and novel therapeutic target.

## Background

Duodenal papilla carcinoma (DPC) is a rare malignancy of the gastrointestinal tract, composing only 1% of all digestive system cancers, with an ascending morbidity year by year [1, 2]. Standard pancreaticoduodenectomy is the predominant treatment for DPC, and 5 year post-surgical survival rate was approximately 50% [3, 4]. However, even with radical surgery, the rate of metastasis is still as high as 25–43% [5]. Furthermore, postoperative complications of pancreaticoduodenectomy is up to 50%, and about1.78% patients need reoperation due to severe complications [6, 7]. Current status in DPC diagnosis and treatment is still far from being satisfactory. Because of the scarcity of this disease, the research about its underling mechanism is limited. The severe postoperative complications and high rate of metastasis urge us to investigate into novel therapeutic targets and prognostic markers for optimizing the postoperative management. Thus, further understanding of the biology and pathogenesis underlying the molecular alterations driving DPC is mandatory.

Aberrant gene expression is a common theme of malignant diseases, corresponding mRNA alterations play pivotal roles in the formation and progress of human cancers [8]. The emergence of high-throughput sequencing technology allows investigators to detect a complete data of the mutational and transcriptional landscape of most tumors, which has improved the understanding of cancers on its molecular mechanisms [9]. However, the low incidence and the scarce of satisfactory samples have dramatically limited our understanding of the biology of tumor formation and treatment in DPC. To date, it was demonstrated that aberrant gene expression and mutation are involved in carcinogenesis and progression of DPC, and several molecular have been recognized as prognostic markers and potential therapy targets. For instance, over expression of the Carcioembryonic Antigen (CEA) and CA19-9 is strongly associated with cell–cell recognition, proliferation, differentiation in DPC [7, 10]. Chemokine CXCL12 and its receptor CXCR4 are also reported to be involved in initiation, invasion, metastasis and prognosis of DPC and even may provide new insight into the targeted therapy [11]. However, mechanism underlying DPC which may provide personalized treatment strategies and prognostic markets is yet to be fully elucidated. At present, RNA-Seq technology combined with bioinformatics analysis enable it a promising way to comprehensively explore the aberrations of mRNA expression across the formation and development of DPC. Investigation into these genes could provide valuable information in understand disease mechanisms which can help to devise optimal treatment and even predict disease relapse.

In the current study, we performed a complicated differential analysis of DPC, trying to identify differentially expressed genes (DEGs) in DPC distinct from those in non-DPC by analyzing datasets obtained from the integrating RNA-seq data, and explore the potential bio-functions by Gene Ontology (GO) and Kyoto Encyclopedia of Genes and Genomes (KEGG) enrichment analyses. Moreover, the protein–protein interaction (PPI) network was mapped to identify the potential interactions between the genes’ products. The current research may provide new insight into the understanding of DPC pathogenesis and the identified hub genes may serve as potential targets for diagnosis and treatment.

## Materials and methods

### Sample preparation and RNA-seq

Three patients underwent radical resection of the primary tumor was included in this study and all samples were recognized as poorly-differentiated DPC by postoperative pathology. DPC tissues and their adjacent normal tissues were collected for transcriptome sequencing analysis and immersed in a cold RNA storage solution (Ambion, USA) immediately after tumors were removed and stored at − 80 °C prior to RNA extraction. This study was in accordance with the Declaration of Helsinki, and all patients were informed about the sample detection and have signed a written informed consent before the surgery. Total RNA isolation for each cancer tissue and corresponding matched normal tissue was carried out using TRIZOL reagent (the RNeasy Mini Kit (Qiagen, Germany)) according to standard RNA extraction protocols. An Agilent 2100 RNA Nano 6000 Assay Kit (Agilent Technologies, CA, USA) was used to qualify the integrity and concentration of total RNA. Later, all the specimens were sent to BGI (The Beijing Genomics Institute) Corporation (Wuhan, China) for further RNA-seq detection and analysis via BGISEQ-500 sequencer.

### Identification of DEGs

DEGs between DPC and non-DPC samples from the obtained data (RNA-seq dataset) were screened using R scripts, furthermore, only the data with |log2FC| ≥1 and *Q* value ≤0.05 were retained as meaningfully DEGs for further analysis. As a result, we identified 110 DEGs and the Dataset was presented in Additional file 1: Table S1.

### GO analysis, enrichment, KEGG pathway analyses

To uncover the functional roles of the screened DEGs acquired by RNA-seq, we performed GO and KEGG enrichment analyses. Based on the DR.TOM system of BGI, the Gene Ontology (GO) enrichment analysis was performed concentrated on the terms biological process (BP), cellular component (CC) and molecular function (MF) and the KEGG pathway analysis was carried out to determine the significant pathways of the DEGs. The threshold of the hypergeometric distribution test for default enrichment results was 0.05.

### Protein–protein interaction (PPI) network analysis and identification of hub genes

To illustrate the protein–protein interaction (PPI) information of the screened DEGs, we mapped the DEGs to the online tool-Search tool for the retrieval of interacting genes (STRING) database, and only interactions enjoyed a minimum required combined score > 0.4 were set as significant. Subsequently, the plug-in MCODE (version 1.5, http://apps.cytoscape.org/apps/mcode) was carried out to identify significant modules of the constructed network. Furthermore, the potential key genes were identified based on the PPI network.

### Quantitative reverse transcriptional PCR (qRT-PCR)

Total RNA of collected samples was isolated using the E.Z.N. ATotal RNA Kit I (Invitrogen) according to the protocol. Complementary DNA (cDNA) was synthesized subsequently by using the Prime Script RT Reagent Kit (Takara Bio, Japan). Real-time PCR was carried out on the ABI 7500 Touch RealTime PCR Detection System (ABI. USA) to measure the expression levels of selected hub genes using the comparative Ct method. Glyceraldehyde-3-phosphate dehydrogenase (GAPDH) was set as a normalization control. The sequences of primers are presented in Additional file 2: Table S2**.**

## Results

### DEGs associated with DPC

**Fig. 1:**
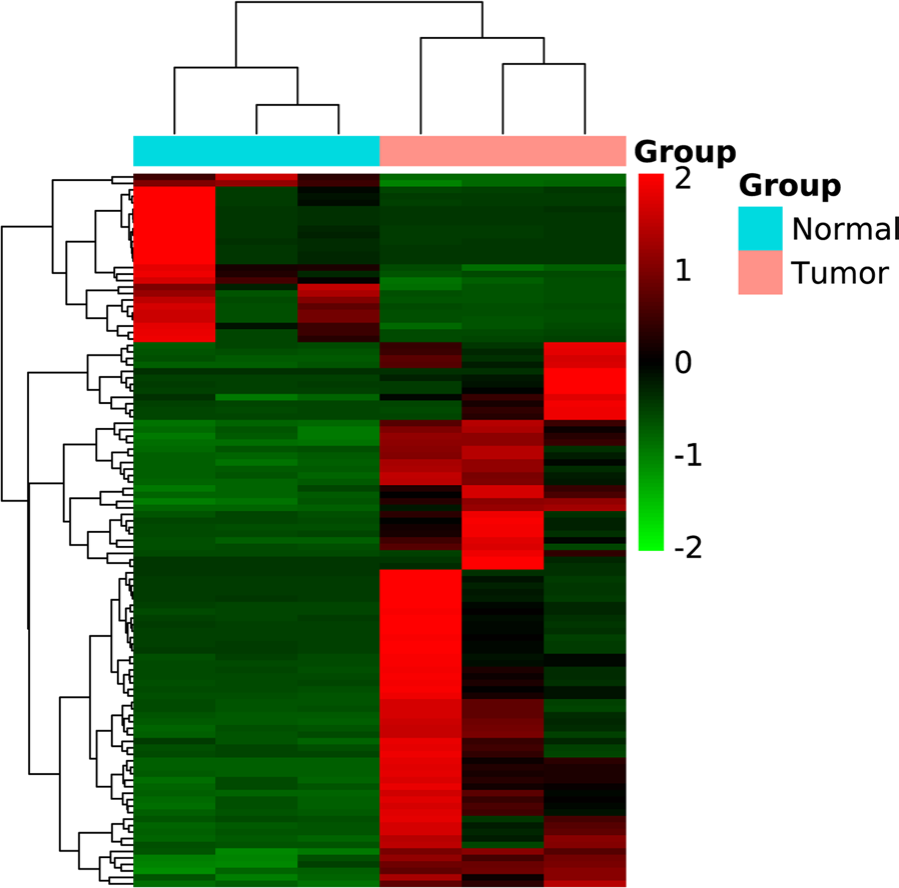
Heat map of the differentially expressed genes in three DPC samples

Paired differential gene expression by RNA-seq analysis revealed that 110 genes were differentially expressed in the 3 pairs of samples. Among the selected DEGS, 84 were upregulated genes and 26 were downregulated genes. Distributions heatmaps of 10 up- and down-regulated DEGs are present in Fig. 1.

### GO enrichment analyses of DEGs

**Fig. 2:**
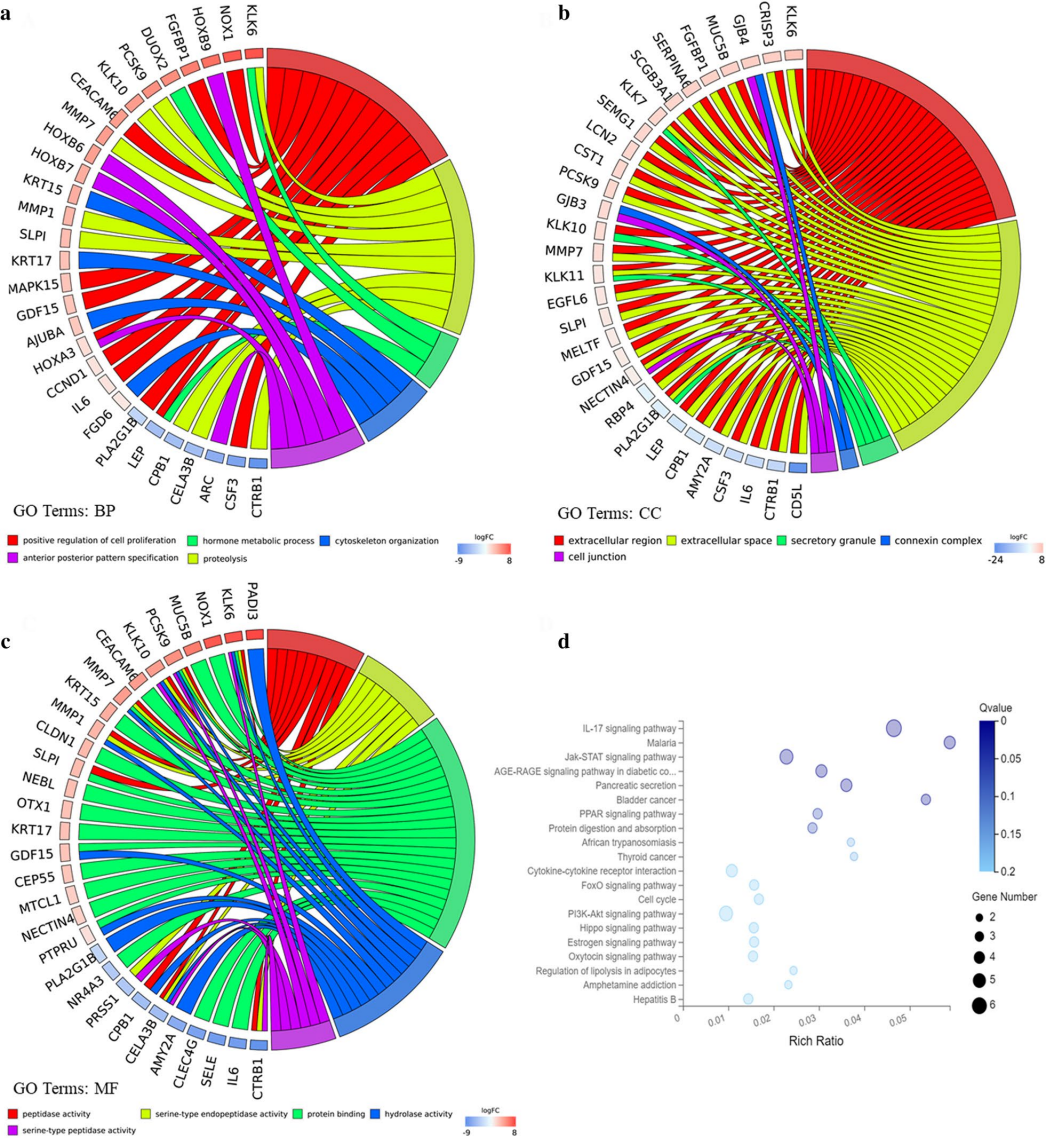
GO and KEGG pathway enrichment analyses for DEGs. **a** Chord plot of GO terms under BP category. **b** Chord plot of GO terms under MF category. **c** Chord plot of GO terms under CC category. **d** Dot plot of KEGG pathway enrichment analyses

To put further insight into the biological functions of the DEGs in human DPC, we performed GO annotations and KEGG pathway enrichment analysis. GO analysis determined several significantly enriched terms, incorporating terms correlated with molecular function (BP), biological process (CC) and cellular component (MF) (Fig. 2a–c). For BP, DEGs were significantly enriched in positive regulation of cell proliferation, hormone metabolic process, cytoskeleton organization, anterior posterior pattern specification, proteolysis. For CC, the DEGs were mainly concerned with extracellular region, extracellular space, secretory granule, connexin complex and cell junction. Moreover, for category MF, the main functions of proteins encoded by the DEGs were peptidase activity, serine-type endopeptidase activity, protein binding, hydrolase activity, serine-type peptidase activity.

### KEGG pathway analysis

Pathway terms with prominent enrichment were figured out and DEGS were opt for the pathway category analysis. It is noteworthy that multiple pathways associated with tumorigenesis were enriched, incorporating IL-17signaling pathway, Jak-STAT signaling pathway, Bladder cancer, PPAR signaling pathway, Cytokine-cytokine receptor interaction, PI3K-Akt signaling pathway, Estrogen signaling pathway. In addition, the DEGs were also enriched in pancreatic secretion, cell cycle, protein digestion and absorption and oxytocin signaling pathway (Fig. 2d).

### Protein–protein interaction (PPI) network analysis and functional module analysis

**Fig.3:**
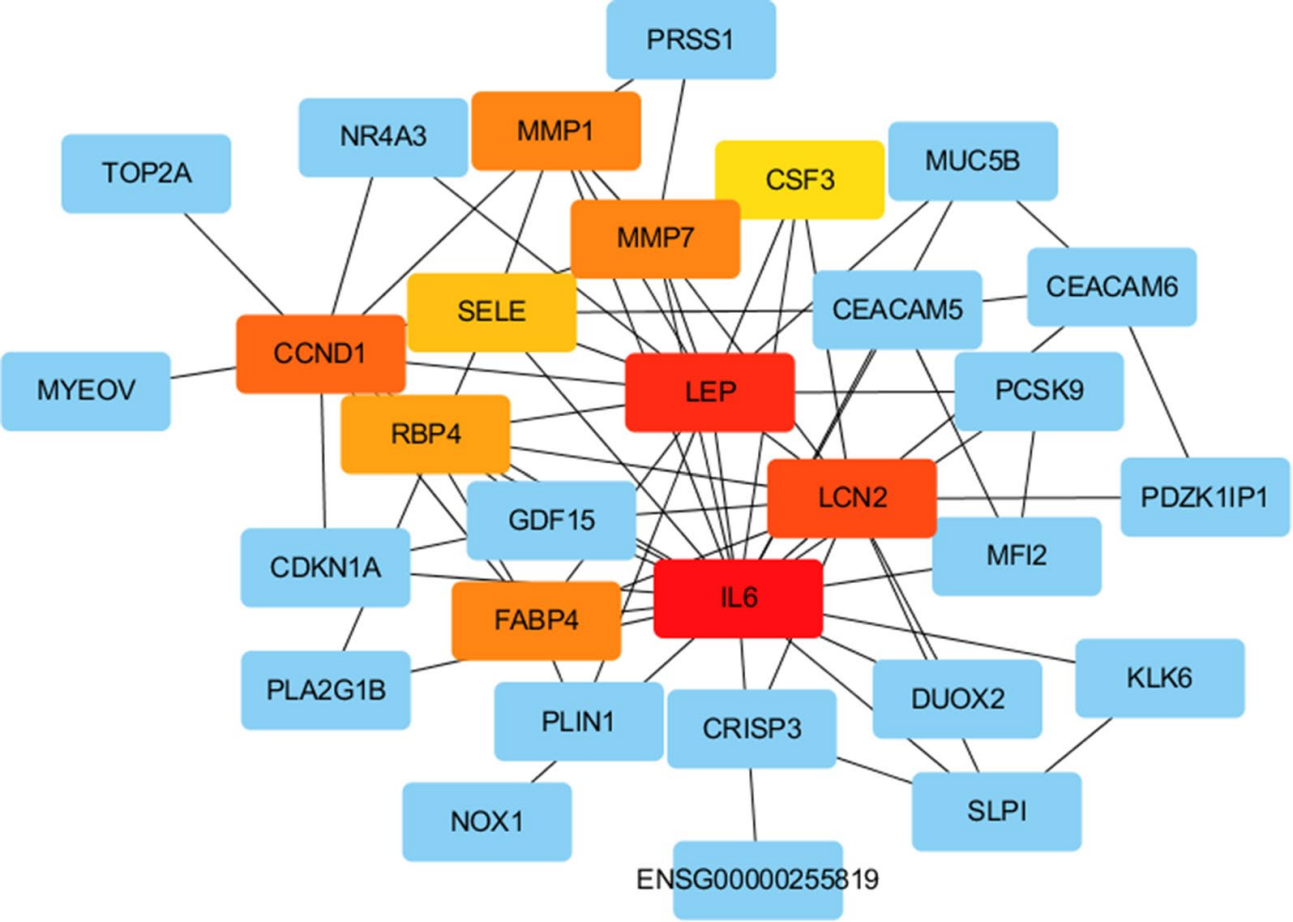
Top 5 hub genes in the PPI network

**Fig. 4:**
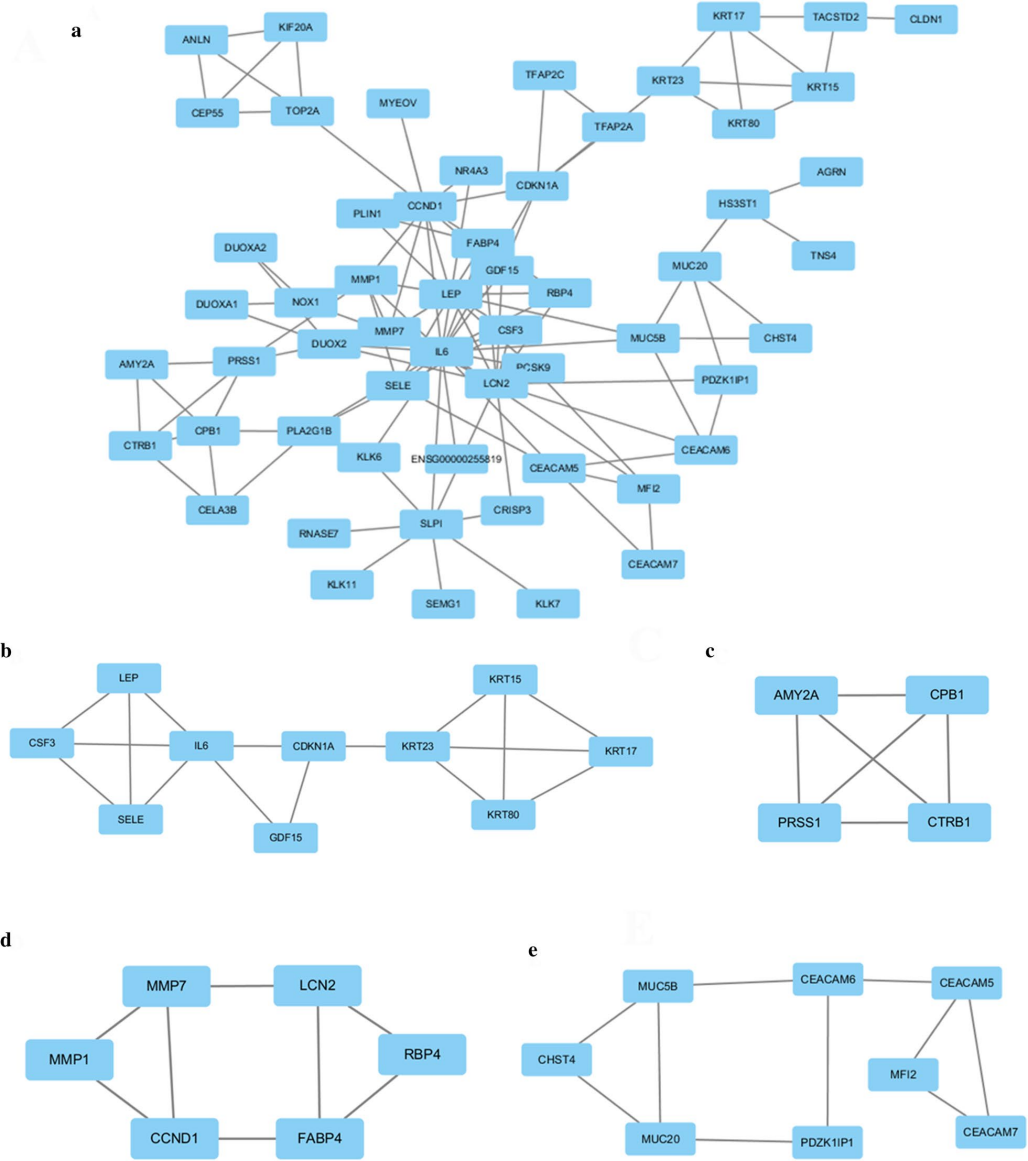
PPI network construction and functional module analysis. The whole PPI network (**a**). Network of functional module 1 (**b**). Network of functional module 2 (**c**). Network of functional module 3 (**d**). Network of functional module 4 (**e**)

In this study, we carried out a multi-step analysis to discover hub genes of DPC, and further investigated their potential biological functions. “Hub genes” are defined as core genes whose expression products are hubs in the PPI network. According to the PPI, five hub genes (IL6, LCN2, FABP4, MMP1 and LEP) were filtered out (Fig. 3). This network consists of convinced interactions from curated databases and those that were experimentally determined. The direct or indirect function interaction of proteins was visualized (Fig. 4a). Moreover, four functional modules were identified through clustering analysis carried out by Cytoscape plug-in MCODE from the whole network (Fig. 4b–e). And that, pathway enrichment analyses for genes of the four modules showed that these modules were mostly involved in Jak-STAT signaling pathway, PI3K-Akt signaling pathway, HIF-1 signaling pathway, Pathways in cancer, p53 signaling pathway, Pancreatic secretion, Pancreas diseases, PPAR signaling pathway, Wnt signaling pathway, Hedgehog signaling pathway, AMPK signaling pathway, IL-17 signaling pathway (Table 1).

**Table 1:** KEGG enrichment analysis of top 4 modules identified from PPI network

Module	KEGG Entry	Description	*p* value	FDR
Module 1	hsa04630	Jak-STAT signaling pathway	8.56E−06	1.50E−08
	hsa04151	PI3K-Akt signaling pathway	3.52E−03	2.93E−05
	hsa04066	HIF-1 signaling pathway	2.77E−02	1.71E−04
	hsa05200	Pathways in cancer	7.68E−03	3.81E−03
	hsa04115	p53 signaling pathway	1.84E−02	1.29E−02
Module 2	hsa04972	Pancreatic secretion	4.30E−11	1.01E−11
		Pancreas diseases	9.17E−04	6.39E−04
Module 3	hsa03320	PPAR signaling pathway	5.82E−05	2.83E−05
	hsa04310	Wnt signaling pathway	2.51E−04	1.22E−04
	hsa04340	Hedgehog signaling pathway	7.32E−03	5.10E−03
	hsa04152	AMPK signaling pathway	1.84E−02	1.28E−02
Module 4	hsa04657	IL-17 signaling pathway	1.90E−02	1.33E−02

### Expression validation

**Fig. 5:**
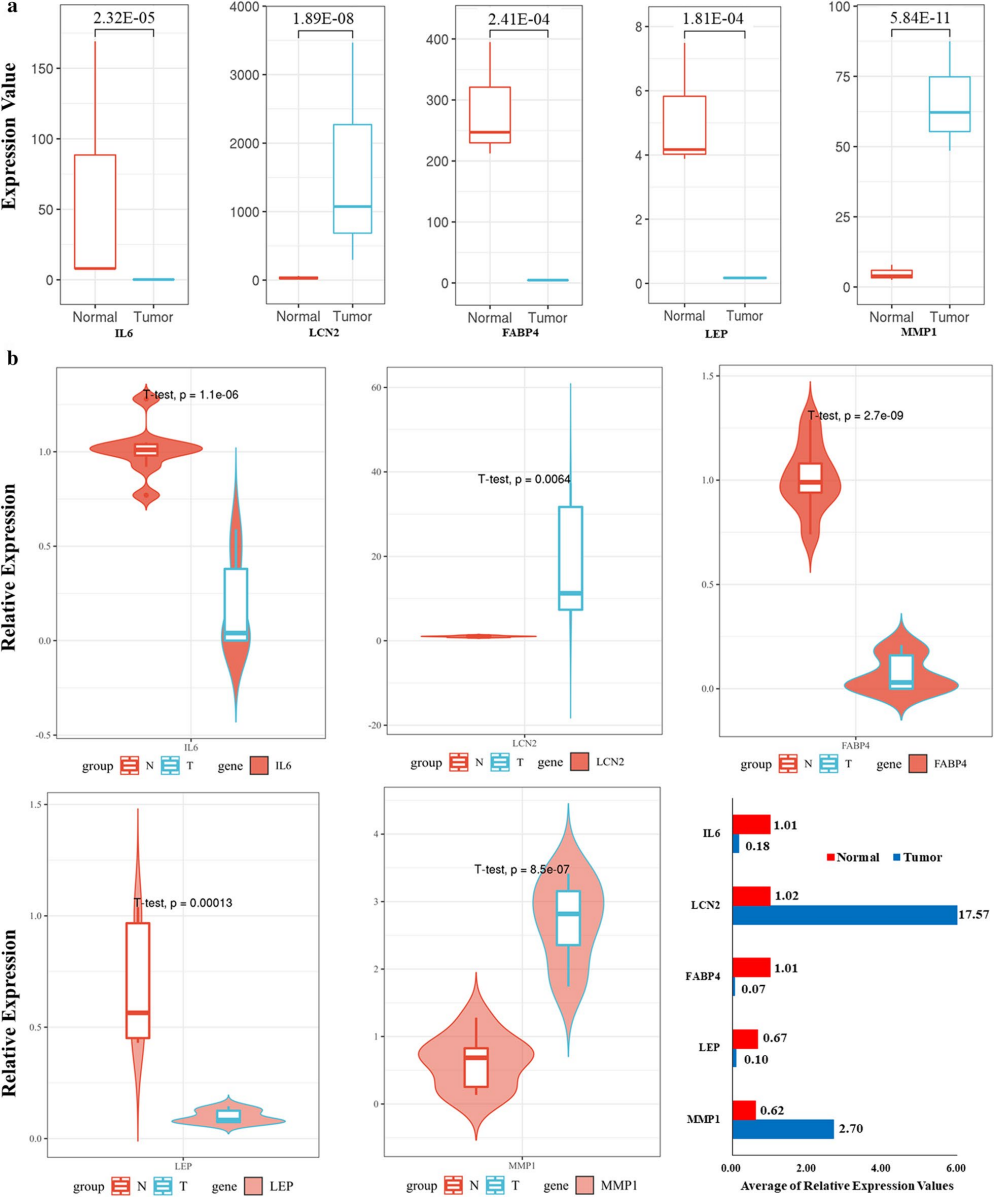
The expression of hub genes in DPC samples by RNA-seq (**a**) and the validation of hub genes expression in DPC and non-DPC clinical samples by qRT-PCR (**b**)

To enhance the accuracy of the RNA-seq data, we validated the expression of hub genes in tumors and matched normal samples. As a result, the relative expression level of IL6, LEP, and FABP4 in DPC samples were significantly lower than in non-DPC samples (*P* < 0.05), and LCN2, MMP1 expression in DPC samples were significantly higher (*P* < 0.05). This result was consistent with the RNA-seq analysis. (Fig. 5).

## Discussion

Tumorigenesis is frequently accompanied by multiple genetic alterations at the transcription level [12]. High metastasis rate of DPC after radical resection has made it a worldwide public health problem. Scarcity of cases of this disease has greatly restricted the recognition of molecular mechanism underlying DPC initiation and progression, and then clinicians are not able to make an early diagnosis as well as design individualized treatment options, this is presumably due to lack of identification of specific biomarkers, either in genes or proteins. Fortunately, RNA-seq technology has provided a possibility for elucidating the mechanism of tumor development at the mRNA level. The combination of bioinformatics and RNA-seq technology have generated a vast amount of gene expression profiles in tumors, and statistical analysis of the identified DEGs could shed light on pathogenesis, and therefore, indicating directions in clinical diagnosis, treatment and prognosis.

In the current cohort, 110 DEGs, including 84 upregulated and 26 downregulated genes, were identified from the RNA-seq analysis results of DPC samples. GO and KEGG enrichment analyses revealed that the DEGs were predominately associated with multiple cancer-related functions and pathways, such as cell proliferation, IL-17signaling pathway, Jak-STAT signaling pathway, PPAR signaling pathway, Cytokine-cytokine receptor interaction and PI3K-Akt signaling pathway. The PPI network was constructed based on the STRING database and module analysis were carried out to further explore functional sub-networks. As a result, IL-6, LCN2, FABP4, LEP, MMP1 were identified as core hub genes in the network and potential therapeutic targets of DPC.

In our cohorts, five hub genes were identified to be associated with a significant risk of DPC pathogenesis and progression. Among these genes, IL-6 has been identified as a hub gene of DPC. Interleukin-6 (IL-6) encodes a cytokine that is produced and secreted by various types of cells including almost all types of tumors which take an active part in proliferation and differentiation of malignant cell [13, 14]. In the tumor microenvironment, IL-6 are mostly secreted by tumor cells as well as tumor-associated macrophages (TAMs), CD4 + T cells, myeloid-derived suppressor cells (MDSCs) and fibroblasts which directly supports tumorigenesis [15]. Molecular evidence has demonstrated that inflammatory signaling pathways are upregulated in gastrointestinal neoplasms, especially NF-κB and IL-6 [16]. In the prostate cancer, the IL-6–STAT3 signal path is involved in protecting against tumor progression via maintaining an intact senescence-inducing ARF–MDM2–p53 tumor suppressor axi [17]. Furthermore, aberrant IL-6 expression is associated with aggressive tumor growth and resistance to therapies in many types of cancer. In our study, IL-6 mRNA was down-regulated and IL-6 was identified to be a core gene of DPC. Therefore, IL-6 could be recommended as a potential target for DPC treatment.

Lipocalin 2 (LCN2) is a secreted glycoprotein of lipocalin superfamily that is an important innate immunity component against bacterial pathogens and various cellular stress [18]. Abnormal expression of LCN2 plays an extremely important role in the epithelial-to-mesenchymal transition process, angiogenesis, and cell migration and invasion in several cancers by involving multiple signaling pathways [19]. A lot of research has indicated that LCN2 is correlated with high-grade malignancy, relapse proneness, metastasis and poor prognosis in epithelial-derived tumors [20]. In our study, LCN2 was selected as critical genes and is significantly upregulated in DPC, which indicates that LCN2 may plays critical roles in initiation and progression of DPC. Combined with the previous studies, there is every reason to believe the tumor-promoter effects of LCN2 on DPC.

For hub gene FABP4, MMP1 and LEP, there have been few reports about their effect on DPC, this is probably because the small number of cases in the previous study. FABP4, also known as aFABP and aP2, is a low-molecular-weight protein that transports LCFAs and other hydrophobic ligands, which takes an active part in the interaction between tumor and adipose tissue [21]. Furthermore, unregulated expression of FABP4 has been recognized as critical indicators of tumor initiation and progression [22]. MMP1 is an interstitial collagenase that is involved in the degradation and proteolytic process of the extracellular matrix. Previous evidence revealed that aberrant expression of MMP1could facilitate cell invasion by the mitogen-activated protein kinase (MAPK) pathway [23]. LEP is derived from adipocytes which is mainly involved in cell energy balance that is considered as a contributor to the development of several gastrointestinal (GI) neoplasms. Since then, assessment of leptin as a modulator of DPC is reasonable. In the current research, the aforementioned genes are differentially expressed in tumor samples of DPC, and their gene products play key roles in protein networks. Based on these findings, we speculate that these genes might play an important role in formation and progression of DPC.

The low incidence and difficulty in obtaining tissue samples greatly limits molecular biology researches into DPC pathogenesis. To the best of our knowledge, our research is the first attempt to carry out the combined transcriptomics and bioinformatic analysis on DPC. Directly RNA -seq of tumor sample and normal tissue sample from the same DPC individual provided relatively reliable and valid data of altered expression of numerous genes at the mRNA level. However, our study has limitations. Firstly, since TCGA database does not provide transcription data for us to identify DPC samples from non-DPC ones, the survival analysis of the identified critical genes was not able to perform to recognize the relationship with prognosis. Therefore, even though this study contributes to the exploration of molecular mechanisms of screened genes, further detailed research is essential to elucidate their prognostic values on DPC. Secondly, correlations between core genes and clinicopathological indicators was not analyzed in this study due to the lack of clinical information of GEO samples. We will solve these deficiencies when we collect enough specimens of DPC.

## Conclusion

In the current research, 110 DEGs between DPC and non-DPC were identified by bioinformatic analysis based on RNA-seq data from biological samples, and five hub genes (IL-6, LCN2, FABP4, MMP1, LEP) were screened out and validated. These genes are closely related to tumorigenesis associated signaling pathways and may play critical roles in DPC progression and even postoperative metastasis. Our results may provide a novel understanding of the pathogenesis of DPC, and the selected genes may be considered as potential therapeutic targets. Further study is needed to investigate into the underlying mechanisms of their effect on DPC.

## Supplementary Information

**Additional file 1: Table S1.** RNA-seq dataset about the 3 DPC patients. A total of 110 DEGs were identified incorporating 84 high-expressed genes and 26 low-expressed genes in DPC tissues compared to adjacent normal samples.

**Additional file 2: Table S2.** The sequences of primers.

## Data Availability

All data generated or analyzed during this study are included in this published article and its supplementary information files**.**
