# Supplementary material for: Identification of critical pathways and potential therapeutic targets in poorly differentiated duodenal papilla adenocarcinoma
Source: Cancer Cell Int. 2021 Jan 6;21:9. doi: 10.1186/s12935-020-01709-7 (PMC7789135; doi:10.1186/s12935-020-01709-7)
Supplement: Supplementary file 1 — Additional file 1: Table S1. RNA-seq dataset about the 3 DPC patients. A total of 110 DEGs were identified incorporating 84 high-expressed genes and 26 low-expressed genes in DPC tissues compared to adjacent normal samples. [file 12935_2020_1709_MOESM1_ESM.pdf]

Supplementary material 1 110 Differentially Expressed Genes

Gene Symbol	Type	Normal Expression	Tumor Expression	Pvalue (tumor / normal)	log2 (tumor / normal)	Change
AMY2A	mRNA	6.913333333	0.083333333	2.54E-04	-6.758615364	down
ARC	mRNA	5.76	0.056666667	1.00E-06	-6.561441462	down
BLK	mRNA	17.55666667	0.49	1.34E-04	-5.160276864	down
CD5L	mRNA	7.696666667	0	1.34E-09	-23.68698311	down
CDKN1A	mRNA	116.1233333	37.06666667	1.34E-04	-1.646382166	down
CELA3B	mRNA	3.183333333	0.09	6.06E-05	-5.178270082	down
CLEC4G	mRNA	45.60666667	0.236666667	1.37E-05	-7.514778099	down
CPB1	mRNA	9.49	0.266666667	1.48E-04	-5.131041959	down
CSF3	mRNA	9.893333333	0.06	5.17E-06	-7.409087373	down
CTRB1	mRNA	11.96333333	0.023333333	7.30E-05	-8.706864637	down
FABP4	mRNA	251.4733333	4.456666667	2.41E-04	-5.843740324	down
HAS1	mRNA	2.653333333	0.053333333	2.35E-04	-5.701287258	down
IL6	mRNA	59.03333333	0.176666667	2.32E-05	-7.991020683	down
IRX3	mRNA	2.633333333	0.113333333	5.63E-05	-4.546407691	down
KLRC4-KLRK1	mRNA	1.526666667	0	2.46E-08	-7.948746926	down
LEP	mRNA	5.513333333	0.173333333	1.81E-04	-5.081319325	down
LRRC55	mRNA	1.943333333	0.03	1.87E-04	-5.982675082	down
NR4A3	mRNA	6.06	0.336666667	1.24E-04	-4.177778358	down
PLA2G1B	mRNA	5.12	0.533333333	2.91E-04	-3.276149666	down
PLIN1	mRNA	15.17333333	0.533333333	2.88E-04	-4.90606832	down
PRSS1	mRNA	30.32666667	1.006666667	1.20E-05	-4.341705447	down
RBP4	mRNA	17.48333333	3.04	5.58E-06	-2.536026952	down
SELE	mRNA	12.97	0.05	8.44E-07	-7.948617749	down
SPIB	mRNA	9.523333333	2.326666667	8.06E-05	-2.062710456	down
SPINK2	mRNA	2.193333333	0.27	9.60E-05	-3.184623263	down
STAB2	mRNA	11.84333333	0.113333333	2.55E-04	-6.720619747	down
MUC5B	mRNA	0.336666667	30.06666667	1.63E-06	6.427683315	up
OTX1	mRNA	0.143333333	1.28	5.39E-05	3.119913871	up
KLK6	mRNA	0.146666667	39.92	3.45E-06	8.037701297	up
MAPK15	mRNA	0.56	4.28	2.24E-04	2.963794248	up
DUOXA2	mRNA	0.32	21.36333333	1.86E-15	6.133849708	up
TOP2A	mRNA	9.123333333	47.64666667	1.45E-04	2.384358134	up
DUOXA1	mRNA	0.096666667	1.066666667	1.55E-04	4.040560488	up
DCDC2	mRNA	0.493333333	5.623333333	4.92E-06	3.529457358	up
CEACAM5	mRNA	12.76666667	366.98	1.71E-05	4.766593036	up
KLK10	mRNA	1.41	54.19	1.99E-10	4.956693014	up
NECTIN4	mRNA	0.77	3.823333333	1.02E-04	2.384579735	up
NEBL	mRNA	1	9.373333333	2.08E-05	3.291711524	up
SPRR1A	mRNA	0	3.846666667	7.46E-09	20.85125723	up
FGD6	mRNA	1.21	2.916666667	2.98E-05	1.25620208	up
SCGB3A1	mRNA	2.846666667	167.0833333	9.08E-06	5.786335988	up
SLPI	mRNA	13.15	141.05	1.30E-07	3.364177051	up
CST1	mRNA	2.003333333	102.09	6.05E-05	5.621474953	up
MUC20	mRNA	12.84333333	98.34666667	7.40E-08	2.932654803	up
HOXB7	mRNA	1.66	27.65	4.86E-05	4.120254012	up
FGFBP1	mRNA	0.196666667	15.53	1.35E-05	6.346033755	up
CCND1	mRNA	13.37333333	36.87666667	2.95E-05	1.446458668	up
ARHGAP8	mRNA	3.883333333	34.66	4.14E-05	3.156525113	up
TFAP2C	mRNA	0.193333333	1.153333333	6.37E-06	2.564047085	up
CEACAM7	mRNA	0.24	10.26	2.54E-07	5.405716589	up
HOXA13	mRNA	0.006666667	3.52	1.69E-04	8.576172749	up
KRTAP5-1	mRNA	0.39	2.923333333	2.56E-04	3.01776876	up

MMP1	mRNA	4.82	66.05666667	5.84E-11	3.721868759	up
YWHAH	mRNA	49.75666667	88.77333333	2.50E-04	0.823191694	up
LOC107984590	mRNA	0.01	1.55	2.76E-04	7.500166433	up
CDH24	mRNA	1.596666667	4.586666667	6.65E-05	1.510159976	up
B4GALNT3	mRNA	0.523333333	7.083333333	1.05E-08	3.763453471	up
KRT23	mRNA	1.283333333	71.22666667	3.25E-05	5.835560256	up
MMP7	mRNA	2.416666667	74.97	2.69E-08	4.876881809	up
SFTA2	mRNA	0.21	8.616666667	2.13E-06	5.326962502	up
SERPINA6	mRNA	0.063333333	5.4	4.73E-05	6.3308095	up
LCN2	mRNA	32.94	1612.813333	1.89E-08	5.621975278	up
ADGRF4	mRNA	0.03	1.666666667	2.78E-07	5.921638609	up
AJUBA	mRNA	0.633333333	4.203333333	2.02E-06	2.747542708	up
PCSK9	mRNA	0.196666667	8.81	9.01E-06	5.518858033	up
PHLDA2	mRNA	3.486666667	37.51333333	5.73E-05	3.462805088	up
KLK7	mRNA	0.22	10.99333333	9.43E-11	5.783636114	up
TNS4	mRNA	0.483333333	19.55333333	4.59E-07	5.333922841	up
GRIN2D	mRNA	0.393333333	7.58	6.78E-05	4.269612444	up
RNASE7	mRNA	0.013333333	1.156666667	1.62E-04	6.132353902	up
ELFN2	mRNA	0.056666667	0.643333333	3.66E-06	3.480517403	up
KRT15	mRNA	0.653333333	10.79666667	7.72E-05	4.090535266	up
DBNDD1	mRNA	2.073333333	10.31333333	7.49E-05	2.34308806	up
CRISP3	mRNA	1.266666667	144.82	7.39E-06	6.795289264	up
TACSTD2	mRNA	1.813333333	71.92333333	9.42E-18	5.335221047	up
EGFL6	mRNA	0.083333333	0.92	7.23E-05	3.435550355	up
KRT17	mRNA	0.46	3.916666667	5.13E-06	3.107184746	up
KIF20A	mRNA	1.72	5.433333333	4.16E-06	1.648737148	up
CACNG4	mRNA	0.163333333	3.206666667	8.88E-10	4.289361387	up
HOXB9	mRNA	0.196666667	23.68666667	2.13E-04	7.001196778	up
HOXB6	mRNA	0.876666667	20.99666667	9.09E-06	4.75475589	up
NOX1	mRNA	0	1.22	1.48E-05	7.877502255	up
SEMG1	mRNA	0.05	2.703333333	2.54E-06	5.715751391	up
TFAP2A	mRNA	0.196666667	2.376666667	1.31E-04	3.593360439	up
DUOX2	mRNA	0.973333333	48.3	4.02E-15	5.671432556	up
CEACAM6	mRNA	20.63	632.78	2.09E-07	4.887118932	up
PADI3	mRNA	0.016666667	2.666666667	4.42E-05	8.476419364	up
GJB5	mRNA	0.056666667	5.376666667	8.11E-07	6.506555493	up
C6orf223	lncRNA	0.186666667	9.37	1.02E-05	5.69317562	up
SLC28A3	mRNA	0.903333333	8.5	9.96E-05	3.134443118	up
PDZK1IP1	mRNA	10.84	151.7866667	1.46E-04	3.812701673	up
MTCL1	mRNA	1.116666667	6.133333333	1.68E-05	2.442279023	up
CA12	mRNA	0.91	29.36333333	6.56E-07	5.069374815	up
GJB4	mRNA	0.04	4.106666667	2.13E-13	6.724543175	up
GDF15	mRNA	3.846666667	29.36666667	1.32E-07	2.95040077	up
ANLN	mRNA	2.196666667	15.55	4.35E-06	2.813025899	up
MYEOV	mRNA	0.23	7.503333333	2.23E-09	5.092648478	up
FAM72C	mRNA	0.623333333	4.676666667	2.09E-04	3.283366125	up
CEP55	mRNA	3.473333333	26.1	1.37E-04	2.901172836	up
HS3ST1	mRNA	3.936666667	11.1	4.95E-05	1.441486704	up
MELTF	mRNA	2.246666667	17.80333333	1.94E-04	2.999136219	up
PTPRU	mRNA	0.77	2.493333333	2.28E-04	1.623761163	up
FIBCD1	mRNA	0.12	8.533333333	1.11E-06	6.191323732	up
AGRN	mRNA	5.683333333	26.92666667	8.70E-05	2.231309492	up
KRT80	mRNA	0.456666667	9.73	6.27E-05	4.415702361	up
CLDN1	mRNA	1.773333333	20.53333333	4.02E-11	3.528549023	up
CHST4	mRNA	0.256666667	14.07333333	2.93E-07	5.769725117	up
GJB3	mRNA	0.463333333	15.76333333	7.25E-11	5.152291272	up
HOXA3	mRNA	1.38	4.543333333	5.78E-05	1.764811531	up
KLK11	mRNA	1.596666667	20.52333333	2.78E-04	3.572125662	up
