# Supplementary material for: Identification of critical pathways and potential therapeutic targets in poorly differentiated duodenal papilla adenocarcinoma
Source: Cancer Cell Int. 2021 Jan 6;21:9. doi: 10.1186/s12935-020-01709-7 (PMC7789135; doi:10.1186/s12935-020-01709-7)
Supplement: Supplementary file 2 — Additional file 2: Table S2. The sequences of primers. [file 12935_2020_1709_MOESM2_ESM.pdf]

Supplementary Material 2 Sequences of primers for qRT-PCR

Gene symbol	Sequences of primers (5'to3‘)
IL6	F: 5'-CAATAACCACCCCTGACC-3'
	R: 5'-GCGCAGAATGAGATGAGTT-3'
LCN2	F: 5'-CAGGGGAAGTGGTATGTGG-3'
	R: 5'-CCTAAACAGGACGGAGGTG-3'
FABP4	F: 5' -AGCACCCTCCTGAAAACTG-3'
	R: 5'-GCAAAGCCCACTCCTACTT-3'
LEP	F: 5'- GAGAGGGCAGAGGGGTAG -3'
	R: 5'- GCTTGTGTTGCTGGGAGT -3'
MMP1	F: 5'- CAGATGCTGAAACCCTGAA-3'
	R: 5'- CAGATGTGTTTGCTCCCA-3'
